# Prognostic value of progesterone receptor in solid pseudopapillary neoplasm of the pancreas: evaluation of a pooled case series

**DOI:** 10.1186/s12876-018-0914-8

**Published:** 2018-12-14

**Authors:** Feiyang Wang, Zibo Meng, Shoukang Li, Yushun Zhang, Heshui Wu

**Affiliations:** 1 Department of General Surgery, Shanghai Jiaotong University Affiliated First People’s Hospital, Shanghai, 200080 China; 2 Department of Pancreatic Surgery, Union Hospital, Tongji Medical College, Huazhong University of Science and Technology, Wuhan, 430022 China; 3 Department of Thoracic Surgery, Union Hospital, Tongji Medical College, Huazhong University of Science and Technology, Wuhan, 430022 China

**Keywords:** Progesterone receptor, Solid pseudopapillary tumor, Pancreas, Prognosis

## Abstract

**Background:**

The role of progesterone receptor (PR) has been reported in a series of pancreatic cysts. However, the relationship between PR and prognosis of solid pseudopapillary neoplasm of the pancreas (SPNP) has not been elucidated so far. The aim of our study was to evaluate the prognostic value of PR in SPNP.

**Methods:**

A total of 76 patients with SPNP treated in our institution from January 2012 to December 2017 were included. Demographic parameters, laboratory data, pathologic information and clinical outcomes were analyzed by the use of survival analysis. In addition, a pooled case series was performed to evaluate the results.

**Results:**

The institutional data included 76 patients (17 male and 59 female) ranging from 8 to 90 years (median, 30 years) in age. Kaplan-Meier survival analysis confirmed negative PR result was significantly associated with poorer disease-free survival (DFS) and disease-specific survival (DSS) (both *P* < 0.001). In the pooled analysis, a total of 62 studies comprising 214 patients with SPNP were included. After multivariable cox analysis, negative PR result remained an independent prognostic factor for SPNP (DFS HR: 14.50, 95% CI: 1.98–106.05, *P* = 0.008; DSS HR: 9.15, 95% CI: 1.89–44.17, *P* = 0.006).

**Conclusion:**

Our results indicated the role of PR in predicting adverse outcome of patients with SPNP and negative PR result may serve as a potential prognostic factor.

**Electronic supplementary material:**

The online version of this article (10.1186/s12876-018-0914-8) contains supplementary material, which is available to authorized users.

## Background

Solid pseudopapillary neoplasm of the pancreas (SPNP), also called Frantz’s tumor, was first described in 1959 [1]. It is a rare pancreatic neoplasm of uncertain lineage, relatively indolent and female predominant, accounting for 1–2% of exocrine pancreatic neoplasms and 5% of cystic pancreatic tumors [2–4]. The World Health Organization currently classifies SPNP as low grade pancreatic malignancy, so excellent survival result is achieved after aggressive surgical resection [5]. The overall survival rate after 5 years surgical resection is about 95–98% [3, 6]. Solid pseudopapillary neoplasm of the pancreas usually show benign in nature, but approximately 10–15% of SPNP demonstrate malignant behavior with adjacent organ invasion, recurrence and metastasis [7, 8]. Albeit with low malignant potential of SPNP, surgical resection is first choice to ensure long term survival even in cases where metastasectomy is required [9, 10].

Owing to the favorable prognosis of SPNP, including the presence of local recurrence and metastasis, predictive factors of survival are difficult to identify. Research have shown the correlation between large tumor size, male sex and younger age with poor prognosis in SPNP [11–14]. While other studies failed to confirm these results [12, 15, 16]. Immunohistochemistry is a common method to diagnosis SPNP in pathology, and some of these parameters are speculated to be the indicators of poor prognosis in different pancreatic cysts. Report found high Ki-67 immunoreactivity, a proliferative index, was related to the recurrence and metastasis of SPNP [17]. The loss of progesterone receptor (PR) expression was also observed in pancreatic neuroendocrine tumor (PanNET) patients with shorter survival time [18]. The role of sex hormone in the origin of SPNP is still enigma, although SPNP always show a tendency to affect young women and positive PR expression [19, 20]. Whilst, more recent studies have not elaborated the effect of PR expression in SPNP prognosis predicting like in PanNET. The aim of this study was to elucidate the prognostic value of PR in SPNP compared with clinicopathological features and immunohistochemistry index such as Ki-67. We also present a pooled case series of published literature for SPNP.

## Methods

### Patients and data collection

We retrospectively analyzed the data from 76 patients admitted in Pancreas Surgery Department of Wuhan Union hospital with pathologically confirmed SPNP from January 2012 to December 2017. All these patients received surgical resection in our hospital. Data collected included patient demographic characteristics, imaging and laboratory data, operative method and pathology through patients’ electronic medical records and paper charts. Patient outcomes were obtained from outpatient records and telephone interview. Patients status were classified as disease free, alive with disease or died of disease and endpoint of follow up was February 28, 2018.

### Pathologic immunohistochemistry for PR

Formalin fixed, paraffin wax embedded tissues were cut into 4 μm sections and mounted on glucose coated slides. Then slides were incubated with mouse anti-human monoclonal PR antibody (1:50, DAKO, Denmark) according to the manufacturer’s guidelines. Two pathologists examined the pathologic slides blinded to the clinical data. The interpretation of PR reactivity was performed as either negative or positive according to the criteria of Reiner et al [21].

### Review of literature

Literature search of PubMed was performed for all articles in English published from 1998 through 2017, using the following Medical Subject Heading (MeSH): Frantz tumor, Solid pseudopapillary tumor, Solid pseudopapillary neoplasm, Pancreas, Pancreatic, Prognosis, Survival and Outcome.

**Fig. 1:**
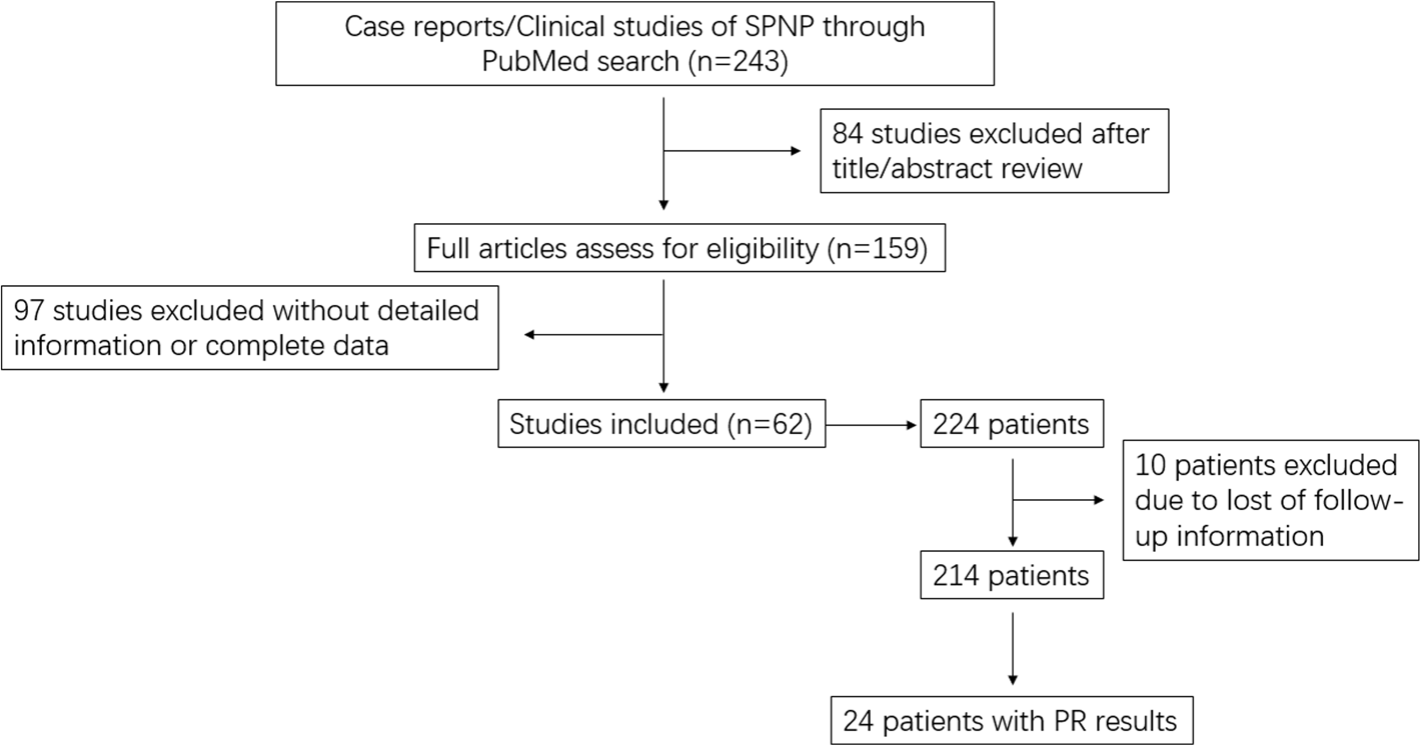
Schematic diagram regarding selection of studies and patient data

Inclusion criteria included original articles, case series and case reports of patients with pathologically confirmed SPNP. Exclusion criteria included reviews, abstracts and studies with limited data such as loss of follow up information. As a result, 62 studies and 214 patients were included. Forty of publications were case reports, and the remaining twenty-two were case series. Schematic diagram regarding selection of studies and patient data was in the Fig. 1. Whenever available, patient data containing demographics, pathology, immunohistochemical results and clinical outcome were extracted. If relevant data were lack in studies, additional information were sought from the corresponding authors. However, only 24 patients with PR result had follow up information, which showed that clinicians did not attach attention to this marker in predicting survival for SPNP.

### Statistical analysis

Statistical Production and Services Solution 19.0 (SPSS 19.0, SPSS) and GraphPad Prism 7 were used in statistical analysis. Continuous data and categorical variables were presented as mean ± standard derivation (SD) and frequency respectively. Categorical variables comparison was performed using chi-square or Fisher exact test and continuous variables were compared by using of Student’s t test. For variables nonparametrically distributed, the Mann Whitney U test was used for comparison. All variables with statistically significant prognostic value in univariable were selected for further multivariable analysis. A COX regression model was used in multivariable analysis. Hazard ratios (HR) and 95% confidence intervals (95% CIs) were presented. Receiver operating characteristic (ROC) curve was performed to determine the optimal discriminator values for continuous variables such as age and tumor size. The optimal cut-off values were determined by Youden index. Evaluation of disease-free survival (DFS) and disease-specific survival (DSS) were obtained by Kaplan-Meier method. *P* value< 0.05 showed a statistically difference.

## Results

### Clinicopathological features of SPNP patients in our cohort

A total of 76 patients with surgical resection in Wuhan Union Hospital and confirmed with SPNP in pathology from January 2012 to December 2017 were included in this study. The clinicopathologic characteristics of 76 patients were presented in Table 1. There were 17 male (22.4%) and 59 female (77.6%), with a median age of 30 years (range, 8–90 years). Forty tumors (52.6%) located in head/neck of the pancreas, and thirty-six tumors (47.4%) located in body/tail. The most common symptom was asymptomatic (47.4%), with tumors occasionally found through physical examination, followed by abdominal pain (38.2%) and mass (6.6%). All tumors were resected, including twenty-three pancreaticoduodenectomy (30.7%), seven duodenum preserving pancreatic head resection (9.3%), thirty distal pancreatectomy with splenectomy (40.0%), five distal pancreatectomy (6.7%), eight central pancreatectomy (10.7%) and two enucleation (2.7%). The tumors ranged from 1.2 to 16 cm in maximum diameter with medium size 5 cm. In immunohistochemistry, all cases showed vimentin (Vim) (71/71, 100%) and β-catenin (73/73, 100%) positive, and most of cases showed PR (68/69, 98.6%), synaptophysin (Syn) (43/70, 61.4%) and CD 10 (68/72, 94.4%) positive. A majority of cases preformed Ki-67 and Chromogranin A (CgA) low proliferation activity (69/71, 97.2%) and negative results (67/71, 94.4%). The mean value of tumor marker in carcinoembryonic antigen (CEA), carbohydrate antigen 19–9 (CA 19–9) and neuron-specific enolase (NSE) were 1.41 ± 0.83, 16.25 ± 31.33 and 21.87 ± 14.64 respectively.

**Table 1:** Demographic and clinicopathological characteristics of 76 patients with SPNP in our cohort

Characteristics			Parameters
Age (Σ = 76)		Mean (y, ±SD)	33.03 ± 17.05
		Median (y, range)	30 (8–90)
Gender (Σ = 76)		Male (%)	17 (22.4%)
		Female (%)	59 (77.6%)
Location (Σ = 76)		Head/Neck (%)	40 (52.6%)
		Body/Tail (%)	36 (47.4%)
Tumor size (Σ = 76)		Mean (cm, ±SD)	5.9 ± 3.3
		Median (cm, range)	5.0 (1.2–16.0)
Symptom (Σ = 76)		Abdominal pain (%)	29 (38.2%)
		Asymptomatic (%)	36 (47.4%)
		Mass (%)	5 (6.6%)
		Other (%)	6 (7.9%)
Immunohistochem istry	Σ71	Vim (Pos itive, %)	71 (100 .0%)
	Σ69	PR (Positive, %)	68 (98.6%)
	Σ70	Syn (Positive, %)	43 (61.4%)
	Σ72	CD10 (Positive, %)	68 (94.4%)
	Σ71	Ki67 (Low proliferation activity, %)	69 (97.2%)
	Σ73	β-catenin (Positive, %)	73 (100.0%)
	Σ71	CgA (Negative, %)	67 (94.4%)
Tumor marker	Σ72	CEA (ng/ ml, ±SD)	1.41 ± 0.83
	Σ72	CA199 (U/ml, ±SD)	16.25 ± 31.33
	Σ45	NSE (ug/L, ±SD)	21.87 ± 14.64
Operation Method (Σ = 75)		Pancreaticoduodenectomy (%)	23 (30.7%)
		Duodenum preserving pancreatic head resection (%)	7 (9.3%)
		Enucleation (%)	2 (2.7%)
		Central pancreatectomy (%)	8 (10.7%)
		Distal pancreatectomy (%)	5 (6.7%)
		Distal pancreatectomy with splenectomy (%)	30 (40.0%)
Follow-up (Σ = 66)		Mean (mo, ±SD)	28.65 ± 19.22
		Median (mo, range)	23.5 (4.0–68.0)
Recurrence/metas tasis (Σ = 66)			6 (9.1%)
Disease related mortality (Σ = 66)			5 (7.6%)

### Clinical outcomes and prognostic factors of SPNP patients in our cohort

Survival data of SPNP patients in our institution were summarized in Table 1. Survival data of Sixty-six patients were eventually selected for analysis because of loss of follow up information in other patients. At a median follow up of 23.5 months (range, 4–68 months), six patients (9.1%) occurred recurrence or metastasis and five patients (7.6%) suffered from SPNP related deaths. The 1-, 3- and 5-year DFS was 97.1, 95.0 and 93.2%. And, the 1-, 3- and 5-year DSS was 98.9, 96.4 and 96.4%, respectively.

**Fig. 2:**
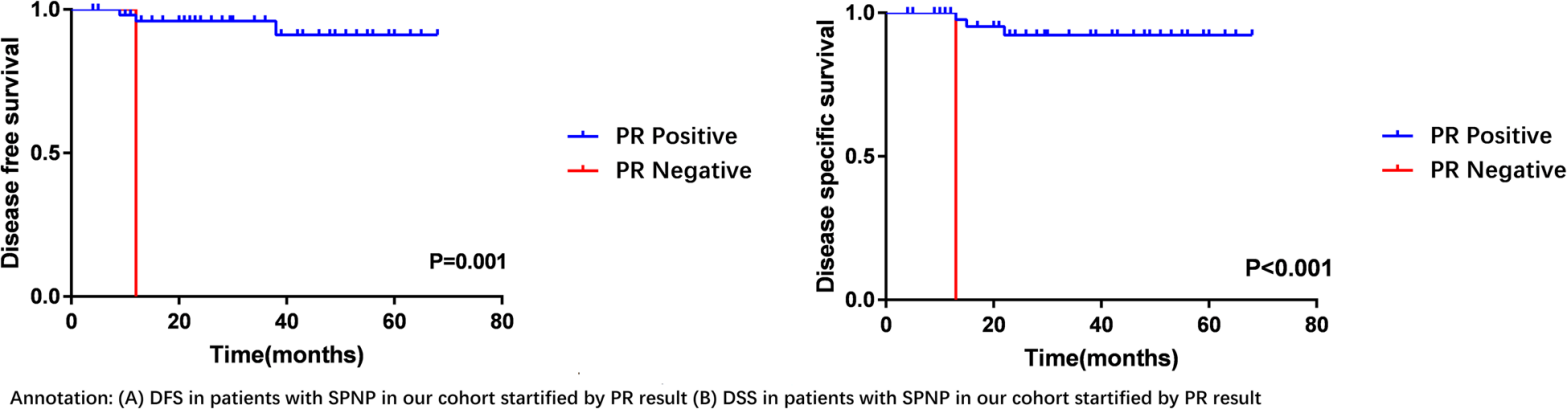
DFS and DSS of SPNP by PR results

**Fig. 3:**
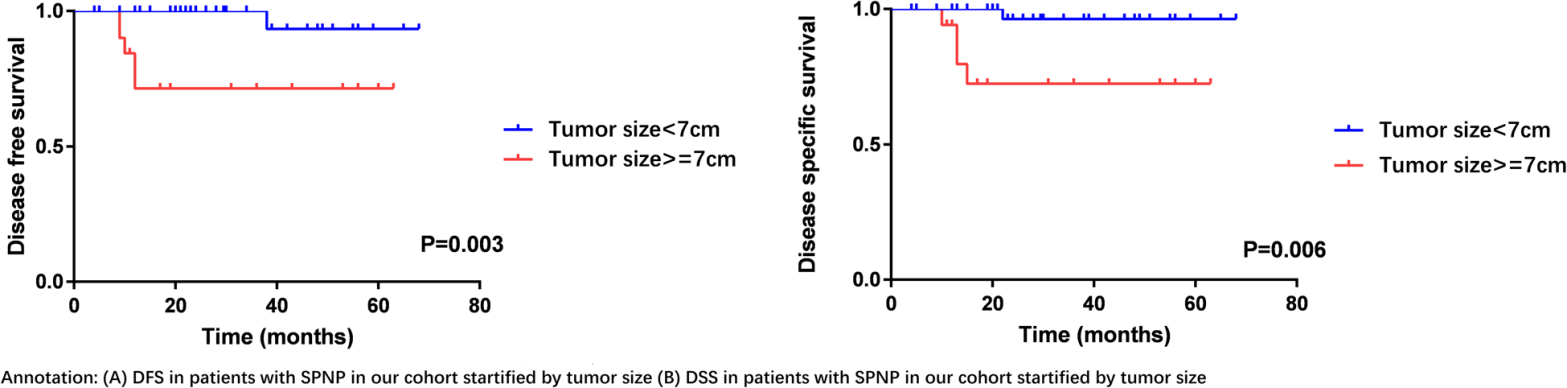
DFS and DSS of SPNP by tumor size

By the use of ROC analysis, the optimal cut-off value of age and tumor size were 40 years and 7 cm. Kaplan-Meier survival analyses and univariable analysis showed that tumor size (≥7 cm) (*P* = 0.003), high Ki-67 proliferation activity (≥5%) (*P* = 0.044) and negative PR result (*P* = 0.001) were associated with the recurrence and metastasis of SPNP. While, large tumor size (≥7 cm) (*P* = 0.006) and negative PR result (*P* < 0.001) were also significantly related to the death of SPNP patients. To the contrary, factors such as age, sex, presence of symptom, tumor location, tumor markers and other immunohistochemical parameters were not associated with the prognosis of SPNP (Additional file 1: Table S1). The DFS and DSS of SPNP patients in our cohort with different PR result and tumor size (≥7 cm) were shown in Figs. 2, 3 by using Kaplan-Meier survival analyses. Multivariable analysis could not be performed here because of the small number of cases.

### PR is an independent prognostic factor for SPNP

According to the literature review, a total of 62 reports and 214 patients with SPNP were included based on our inclusion criteria. The demographic and clinicopathology features of 214 patients were summarized in Additional file 1: Table S2. Combined with 66 SPNP patients in our cohort with follow up information, there were eventually 280 patients in the following prognostic analyses. In the univariable cox regression analysis, we observed age (≥40 years) (*P* = 0.03), high Ki-67 proliferation activity (≥5%) (*P* = 0.006) and negative PR results (*P* < 0.001) were significantly correlated with the incidence of recurrence or metastasis of SPNP. Whilst, tumor size (≥7 cm) (*P* = 0.018) and negative PR result (*P* < 0.001) were related with the death of SPNP. Then, these characteristics showed statistical difference (*P* < 0.05) above were performed in multivariable analysis. The PR result could be an independent prognostic factor of DFS and DSS in SPNP patients (DFS HR: 14.50, 95% CI: 1.98–106.05, *P* = 0.008; DSS HR: 9.15, 95% CI: 1.89–44.17, *P* = 0.006) (Table 2).

**Table 2:** Prognostic factors for DFS and DSS in patients with SPNP according to cox regression models

	Univariate COX model			Multivariate COX model		
Prognostic factors	β	Hazard ratio (95%CI)	*P* value	β	Hazard ratio (95%CI)	*P* value
DFS (*n* = 280)						
Age (≥40 y)	0.982	2.67 (1.10–6.48)	0.030	0.536	1.71 (0.38–7.79)	0.872
Gender (male)	−0.615	0.54 (0.18–1.59)	0.265			
Location (Head/Neck)	0.399	1.49 (0.64–3.45)	0.351			
Tumor size (≥7 cm)	0.809	2.25 (0.87–5.77)	0.093			
*Ki67 (≥5%)	1.770	5.87 (1.65–20.95)	0.006	0.2 93	1.34 (0.19–9.68)	0.771
*PR negative)	2.962	19.34 (5.08–73.61)	< 0.001	2.674	14.50 (1.98–106.05)	0.008
DSS (*n* = 280)						
Age (≥40 y)	0.454	1.58 (0.41–6.09)	0.511			
Gender (male)	−1.295	0.27 (0.07–1.06)	0.061			
Location (Head/Neck)	−0.067	0.94 (0.27–3.23)	0.916			
Tumor size (≥7 cm)	1.878	6.54 (1.39–30.82)	0.018	1.7 16	5.56 (0.60–51.19)	0.130
*Ki67 (≥5%)	1.214	3.37 (0.65–17.48)	0.149			
*PR (negative)	2.800	16.45 (3.66–73.87)	< 0.001	2.2 13	9.15 (1.89–44.17)	0.006

*Number of SPN patients with Ki67 and PR results are 82 and 83 respectively

**Fig. 4:**
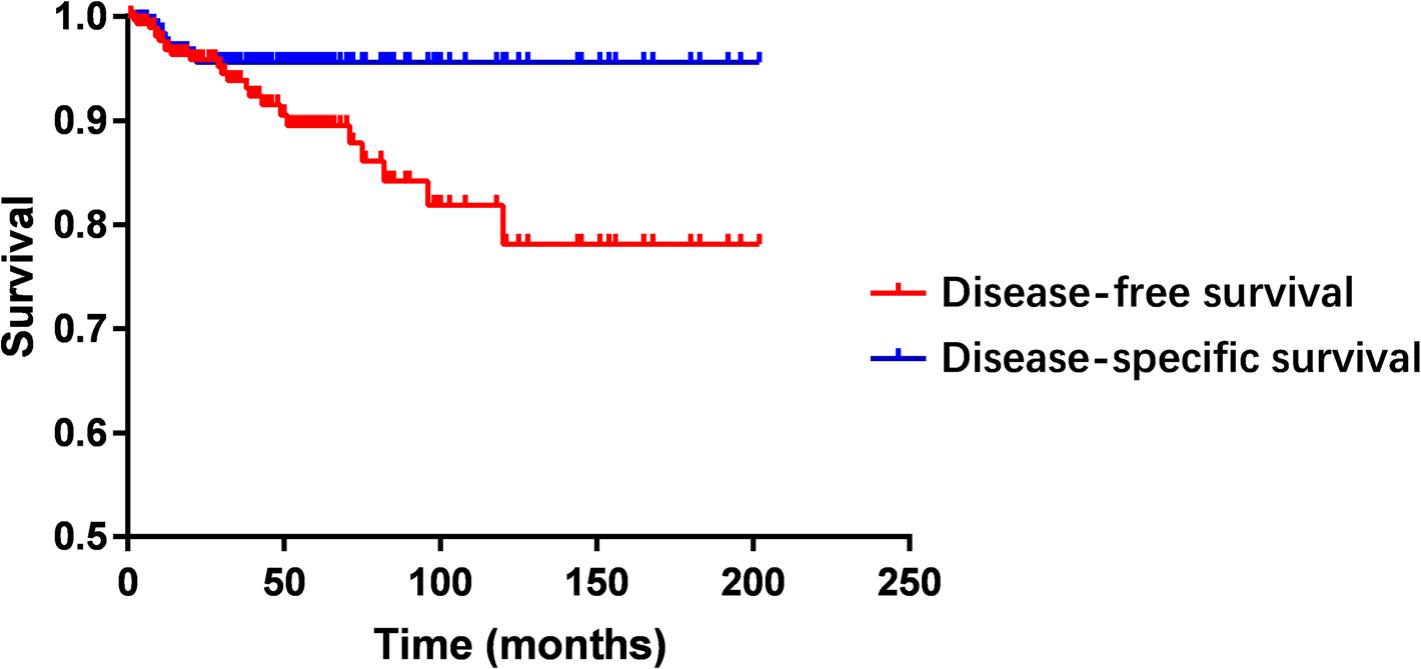
Kaplan Meier survival curves of DFS and DSS in pooled analysis

**Fig. 5:**
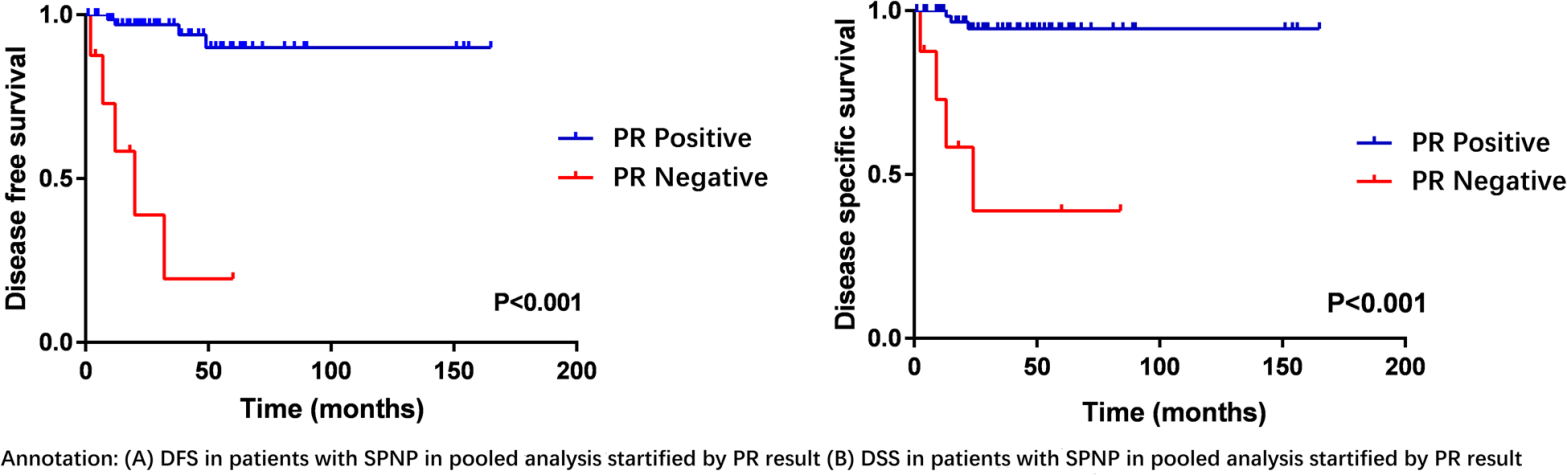
DFS and DSS of SPNP by PR results in pooled analysis

Kaplan-Meier curves of DFS and DSS for patients included in the pooled analysis were shown in Fig. 4. The DFS and DSS of SPNP patients according to PR results were shown in Fig. 5. Negative PR result was associated with poorer DFS and DSS (*P* < 0.001).

### Comparison of clinicopathological parameters between SPNP with different PR results

The clinicopathological features of 93 SPNP patients with PR results were compared in the Table 3 according to the different PR results. The results showed that PR results were significantly related to the tumor size (*P* = 0.01) and Ki-67 (*P* < 0001). And the clinical outcomes of SPNP patients with PR negative result were worse than that of PR positive patients (*P* < 0.001). Therefore, negative PR result was a risk factor for prognosis of SPNP patients.

**Table 3:** Comparison of clinicopathological parameters between SPN with different PR results

Characteristics		PR (+)	PR (−)	*P* value
Age (Σ = 93)		85	8	0.091
	Mean (y, ±SD)	32.85 ± 16.71	44.13 ± 17.61	
	Median (y, range)	32 (8–90)	43 (20–71)	
Gender (Σ = 93)		85	8	0.194
	Male (%)	15 (17.6%)	0 (0.0%)	
	Female (%)	70 (82.4%)	8 (100.0%)	
Location (Σ = 93)		85	8	0.924
	Head/Neck (%)	44 (51.8%)	4 (50.0%)	
	Body/Tail (%)	41 (48.2%)	4 (50.0%)	
Tumor Size (Σ = 92)		84	8	0.010
	Mean (cm, ±SD)	5.95 ± 3.25	9.99 ± 4.29	
	Median (cm, range)	5.0 (1.2–16.0)	11.0 (2.2–15.5)	
Immunohistochemistry				
	Syn (Positive, %) Σ = 93	47/85 (55.3%)	4/8 (50.0%)	0.774
	CD10 (Positive, %) Σ = 79	68/74 (91.9%)	5/5 (100.0%)	0.508
	Ki67 (Low proliferation activity, %) Σ = 75	65/69 (94.2%)	3/6 (50.0%)	< 0.001
	CgA (Negative, %) Σ = 91	75/84 (89.3%)	7/7 (100.0%)	0.362
Recurrence/metas tasis (Σ = 83)		4/75 (5.3%)	5/8 (62.5%)	< 0.001
Disease related mortality (Σ = 83)		3/75 (4.0%)	4/8 (50.0%)	< 0.001

## Discussion

With advances in imaging strategies, the number of detected SPNP was obviously increased in the last decades. More and more research about SPNP were also reported. As a neoplasm with low malignant potential, SPNP usually have a good prognosis after aggressive surgical resection, while further observations found some cases showed a short survival time when recurrence or metastasis happened. Therefore, identifying SPNP that have the potential for malignancy seems particularly important. Researchers have tried to find markers to predict the malignant behavior of SPNP for many years, and contradictory results are reported constantly [11–16]. In this study, we retrospectively analyzed the relationship between clinicopathologic features and clinical outcomes in our institution and a pooled case series. Our result showed that DFS and DSS were significantly shorter in patients with negative PR result and PR was classified as an independent prognostic factor for SPNP after multivariable analysis. To the best of our knowledge, this is the first study to evaluate that negative PR result could effectively predict a poorer prognosis of SPNP patients after surgical resection.

Progesterone receptor, the effector of progesterone, is widely distributed in reproductive system, immune tissue, cardiomyocytes, brain, lung and other organs [22–24]. As a neuroendocrine organ, pancreas also show PR expression in normal pancreatic islets suggesting a possible role for progesterone in pancreatic islets function [25, 26]. A hormonal influence on pathogenesis of SPNP has been postulated in view of its high prevalence in young women. Some previous research attempted to study the role of female sex hormone in SPNP, but conflicting results emerged. PR is consistently positive in SPNP irrespective of sex and hormonal changes, while immunolabeling for estrogen receptor is negative [20, 27]. It is still a mystery whether sex hormone participate in the origin of SPNP. Of note, studies have indicated that PR has prognostic value in many other pancreatic malignancies such as PanNET [18, 28]. They found the loss of PR expression can provide information on shorter DFS in PanNET patients. We know that the activation of PR can have an inhibitory effect on cell proliferation, differentiation and tumorigenesis under the change of gene expression, post-translational modification of proteins and intracellular Ca^2+^ level [29–31]. And in the research of Jeannelyn [32], they found PR play a crucial role in advanced PanNET through the regulation of PI3K-AKT pathway. All above may explain how PR could be the prognostic factor of some malignancies, but the mechanism of PR in predicting SPNP prognosis need further research.

Previous studies have suggested that some clinicopathologic features such as tumor size, younger age and male sex could indicate the malignant potential of SPNP [11, 12, 14]. However, we found there were a shorter DFS and DSS when tumor size ≥7 cm in our institution, but only DSS was related to tumor size in the pooled series. Kang [14] et al. considered tumor size larger than 5 cm showed a malignant potential of SPNP. And in immunohistochemistry, high Ki-67 proliferative activity showed a significant correlation with DFS in both of our institution and pooled series, but not with DSS. In Yang’s study [17], a Ki-67 index ≥4% remained associated with poorer prognosis of patients with SPNP. We also observed age ≥ 40 years was a risk factor of short DFS in univariable analysis. In addition to these parameters, we failed to identify other clinicopathological features in predicting prognosis of SPNP. The reason for different or conflicting results may lie in the fact that some studies used blurred criteria of malignancy or small sample size. Meanwhile, clinicians attach more attention on this kind of tumor in recent decades because of the improvement of imaging and pathology method, so more studies about SPNP were reported.

Due to the recurrence or metastasis of some SPNP after surgical resection, the World Health Organization classified SPNP as low-grade malignant tumor with malignant features of surrounding tissue invasion, perineural invasion, vascular invasion on microscopic pathology and metastasis [33]. Therefore, some studies regarded these malignant performances including muscular vessel invasion, adjacent organ or lymph node invasion and distant metastasis at diagnosis were the prognostic factors of SPNP [34–36]. While, these performances always present late progress of SPNP with poor prognosis, we need a predictive factor on prognosis of SPNP without malignant features at relatively early stage.

## Conclusion

This study highlights the potential prognostic value of PR in patients with SPNP, which may help in risk stratification according to the surgical pathologic report. Although SPNP usually has a good clinical outcome, long term follow-up is necessary because of possible recurrence or metastasis. These patients should be scheduled every 1–2 months follow up visits during the first year after surgery and every 3–6 month for years after [17]. And the effect of adjuvant chemotherapy for high risk SPNP need further confirm. There are several limitations in this study. As a retrospective study, the role of PR in SPNP requires to be investigated in a prospective validation study. The small sample size and short length of follow up precluded the difference between all other effective marker. Multicenter prospective studies with large sample size and long follow up are necessary in the future. What’s more, we did not notice the effect of adjuvant chemotherapy in this study. Nevertheless, pooled analysis is essential for prediction of prognosis of rare disease. The methodology of our study could be reliable [37, 38].

## Additional file

Additional file 1: **Table S1.** Univariable cox regression model of DFS and DSS for patients with SPNP in our cohort. **Table S2.** Demographic and clinicopathological characteristics of 214 patients with SPNP in literature review. (PDF 166 kb)

## Abbreviations

CA19–9: Carbohydrate antigen 19–9
CEA: Carcinoembryonic antigen
CgA: Chromogranin A
DFS: Disease-free survival
DSS: Disease-specific survival
MeSH: Medical Subject Heading
NSE: Neuron-specific enolase
PanNET: Pancreatic neuroendocrine tumor
PR: Progesterone receptor
SPNP: Solid pseudopapillary neoplasm of the pancreas
Syn: Synaptophysin
Vim: Vimentin
