# Supplementary material for: Prognostic value of progesterone receptor in solid pseudopapillary neoplasm of the pancreas: evaluation of a pooled case series
Source: BMC Gastroenterol. 2018 Dec 14;18:187. doi: 10.1186/s12876-018-0914-8 (PMC6295102; doi:10.1186/s12876-018-0914-8)
Supplement: Supplementary file 1 — Table S1. Univariable cox regression model of DFS and DSS for patients with SPNP in our cohort. Table S2. Demographic and clinicopathological characteristics of 214 patients with SPNP in literature review. (PDF 166 kb) [file 12876_2018_914_MOESM1_ESM.pdf]

Supplementary Table I. Univariable cox regression model of DFS and DSS for patients with SPNP in our cohort

Prognostic factors	β	Hazard ratio（95%CI）	*P value*
**DFS（n=66）**			
Age（≥40 y）	-0.234	0.79（0.14-4.39）	0.788
Gender（male）	0.432	1.54（0.18-13.23）	0.694
Location（Head/Neck）	-0.387	0.68（0.12-3.71）	0.655
Tumor size（≥7cm）	2.517	12.39（1.44-106.50）	**0.003**
*Ki67（≥5%）	2.292	9.90（1.07-91.78）	**0.044**
*PR（negative）	3.176	23.95（2.17-264.31）	**0.001**
**DSS（n=66）**			
Age（≥40 y）	-0.883	0.41（0.46-3.70）	0.430
Gender（male）	0.152	1.16（0.13-10.42）	0.892
Location（Head/Neck）	-0.045	0.96（0.16-5.73）	0.961
Tumor size（≥7cm）	2.440	11.47（1.28-102.95）	**0.006**
*Ki67（≥5%）	-3.039	0.06（0.01-198.30）	0.748
*PR（negative）	3.847	46.85（2.93-750.91）	**<0.001**

* Number of SPN patients with Ki67 and PR results are 61 and 59 respectively

Factors such as presence of symptom, tumor markers and other immunohistochemical parameters did not show significantly correlation with DFS and DSS (Data not shown)

Supplementary Table II. Demographic and clinicopathological characteristics of 214 patients with SPNP in literature review

Characteristics			Parameters
**Age** (Σ=214)		Mean（y, ±SD)	24.84±15.04
		Median（y, range)	18（7-79）
**Gender** (Σ=214)		Male（%）	17（7.9%）
		Female（%）	197（92.1%）
**Race** (Σ=214)		Caucasoid（%）	136（63.6%）
		Mongoloid（%）	74（34.6%）
		Black（%）	4（1.9%）
**Location** (Σ=208)		Head/Neck（%）	95（45.7%）
		Body/Tail（%）	113（54.3%）
**Tumor size** (Σ=190)		Mean（cm, ±SD)	6.62±3.49
		Median（cm, range)	6（1-18）
**Symptom** (Σ=144)		Abdominal pain（%）	66（45.8%）
		Asymptomatic（%）	32（22.2%）
		Mass（%）	27（18.8%）
		Other（%）	19（13.2%）
**Immunohistochemistry**	Σ35	Vim（Positive, %）	35（100.0%）
	Σ24	PR（Positive, %）	17（70.8%）
	Σ43	Syn（Positive, %）	12（27.9%）
	Σ15	CD10（Positive, %）	13（86.7%）
	Σ11	Ki67（Low proliferation activity, %）	5（45.5%）
	Σ9	β-catenin（Positive, %）	9（100.0%）
	Σ39	CgA（Negative, %）	32（82.1%）
**Operation Method** (Σ=204）		Pancreaticoduodenectomy（%）	61（29.9%）
		Duodenum preserving pancreatic head resection（%）	10（4.9%）
		Total pancreatectomy（%）	4（2.0%）
		Enucleation（%）	17（8.3%）
		Central pancreatectomy（%）	19（9.3%）
		Distal pancreatectomy（%）	50（24.5%）
		Distal pancreatectomy with splenectomy（%）	43（21.1%）
**Follow-up** (Σ=214）		Mean（mo, ±SD)	51.92±48.87
		Median（mo, range)	36（1-205）

**Recurrence/metastasis** (Σ=214）	17（7.9%）
**Disease related mortality** (Σ=214）	5（2.3%）
